# Will the Increasing of Anthropogenic Pressures Reduce the Biopotential Value of Sponges?

**DOI:** 10.1155/2015/734385

**Published:** 2015-09-20

**Authors:** Hedi Indra Januar, Asri Pratitis, Aditya Bramandito

**Affiliations:** ^1^Indonesian Research and Development Center for Marine and Fisheries Products Processing and Biotechnology, KS Tubun Petamburan VI Street, Slipi, Central Jakarta 10260, Indonesia; ^2^Department of Marine Science, Faculty of Fisheries and Marine Science, Bogor Agricultural University, Kampus IPB, Darmaga Raya Street, Bogor 16680, Indonesia

## Abstract

Production of bioactive compounds from marine benthic organisms is suggested to relate ecologically with environment. However, anthropogenic pressures cause a considerable damage to coral reefs environment. This research aimed to define the pattern sponges biopotential values at the increasing of anthropogenic pressures to coral reef environment. Three representative sponges were selected (*Theonella* sp., *Hyrtios* sp., and *Niphates* sp.) and study had been conducted in Hoga Island, Indonesia, to define the relationship between seawater variables (DO, pH, phosphate, and ammonia ions), sponges spatial competition, and their bioactivity level (Brine Shrimp Lethality Test). The study showed anthropogenic pressures affect the reef environment, as abiotic cover was increased and eutrophication was detected at the site closer to the run-off domesticated area. Statistical multivariate analyses revealed sponges spatial competition was significantly different (*P* < 0.05) between groups of high, moderate, and low bioactivity level. Abiotic cover was detected as the major factor (36.19%) contributed to the differences and also the most discriminant factor distinguishing sponges spatial competition in the groups of bioactivity level (93.91%). These results showed the increasing anthropogenic pressures may result in a higher abiotic area and may directly be a consequence to the lower production of bioactive compounds in sponges.

## 1. Introduction

It is generally accepted that competition for space is a major ecological reason of sponges to produce their bioactive compounds. The occurrence of bioactive compounds in sponges is usually correlated with their attempt to invade or maintain living space in benthic area against competitors [1–5]. The balances of benthic competition in healthy coral reef ecosystem have been considered to stimulate the production of certain chemical defense substances in marine benthic organism. This may mean the diversity of coral community plays an important role in the production of bioactive compounds in sponges. Therefore, it is potential to find new chemical bioactive compounds from sponges in dense benthic communities, such as in the Indonesian CTI (Coral Triangle Initiative) area [6–8].

However, the increasing of anthropogenic pressures, that is, coral mining, destructive fishing activities, and aquatic pollution, has been causing considerable damage to the structure of coral reef community [9–12]. The diversity of coral community tends to be shifted as the anthropogenic pressures increase. As a consequence, it raises a question about how this shifting affects the production of bioactive compounds in sponges. Although the effect of anthropogenic pressures on coral community was already demonstrated in many laboratory and in situ experiments, there is little known about how these pressures affect the biopotential values in sponges. This paper will present the possible correlation between bioactivity variation at metabolites extracted from sponges and benthic competition that is pressured by anthropogenic. This study had been conducted in Hoga Island, one of the most interesting coral reef communities in CTI area at Wakatobi Marine National Park, which was reported to possess approximately 71 species of sponges [13]. The study focused on three species of sponges:* Theonella* sp.,* Niphates* sp., and* Hyrtios* sp. The Brine Shrimp Lethality Test (BSLT) is used as the bioassay system, based on its general relation to cytotoxic activity [14], which is in line with the allelopathic activity needed by sponges to win spatial competition in benthic area.

## 2. Materials and Methods

### 2.1. Study Sites

**Figure 1:**
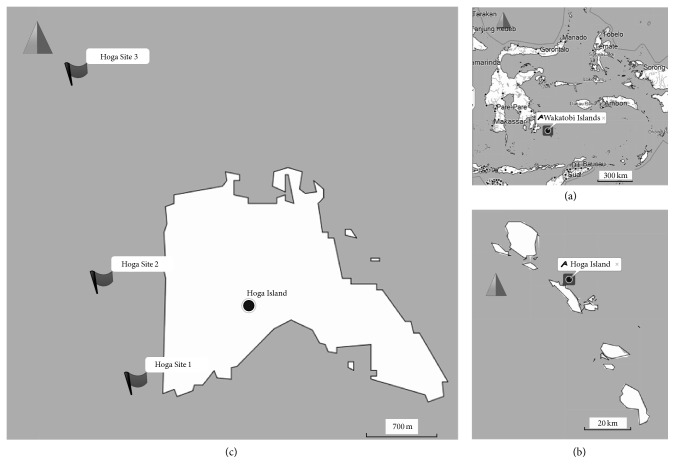
Sampling sites at Indonesian CTI region (a) at Wakatobi Islands (b), on the western side of Hoga Island (c).

The area of study was at the western side of Hoga Island, South East Sulawesi, Indonesia (Figure 1). This island is located at the central part of Wakatobi Islands National Park, one of the Indonesian marine conservation areas. Three sampling sites were selected based on a preliminary Manta Tow study about the coral cover surrounding this island. Visual observation showed that the reef at southern part of this island was highly affected by anthropogenic pressures. Recreational facilities development, such as seaport, and other various anthropogenic might be contributed to coral cover in this area. Enormous plastic waste along the southern beach was noted, which indicates anthropogenic run-off and stresses the coral reef of Hoga Island. Site 1 (S5°28.533′, E123°45.524′) was selected near the small harbor and the recreational facilities in Hoga Island; meanwhile Sites 2 (S5°28.012′, E123°45.338′) and 3 (S5°26.894′, E123°45.200′) were selected further away to the north in order to gain the gradient effects of anthropogenic stressors. The precise locations of the sites were recorded employing a Garmin eTrex 10 GPS and plotted to OpenSeaMap chart with Garmin Basecamp 4.4.6.

### 2.2. Coral Cover and Water Quality Analysis

Coral cover at each selected site was analyzed at 4–6 m depth by employing three 30 m long line intercept transects (LIT). Underwater photographs were taken using a digital camera every meter on both sides of the transect line with a 0.5 × 0.5 m quadrant frame. The analysis had been conducted by Coral Point Count software using a 50-random-distributed-point count methodology [15]. The categories in transect were sponges, hard coral, soft coral, algae, rubble, and sand. Meanwhile, local coral cover at each sponge sample (0.5 × 0.5 m quadrant frame with sponge sample in the center of the frame) was also calculated by the same methodology. The local coral cover for each sponge was conducted to analyze the approximate spatial competitor. The competition was determined by comparing the estimation of the sponge cover relative to other major components on the reefs benthos [16]. Moreover, at each sampling site, water samples were taken in three replicates and twice a day (at low and high tidal). Analysis of PO_4_, NH_3_, DO, and pH was immediately carried out on board above the sampling site using a portable colorimeter Hach DR-890 and a rugged probe DO and pH-meter.

### 2.3. Sponge Preservation and Bioactivity Testing

From each site, 4 replicates of* Theonella* sp.,* Niphates* sp., and* Hyrtios* sp. were collected from their habitat at 5–7 m depth. Sampling of the organisms was carried out with a careful inspection of their morphology similarities, to avoid misleading results due to species variation. Approximately, 5 g of sponge was harvested and preserved with 20 mL of PA grade methanol (JT Baker). All of the samples were kept in dark-brown vials and placed in iced cool box. In the laboratory, samples were exhaustively extracted with methanol and dried with rotary evaporator and concentrator, to yield the crude methanolic extracts that were used for bioactivity study. Each sample voucher was preserved in Biotechnology Laboratory, Indonesian Research and Development Center for Marine and Fisheries Products Processing. In the lab, crude methanolic extracts of sponges were prepared in 100 ppm and subjected to Brine Shrimp Lethality Test. Extract was considered active when it has LC_50_ of more than 50% in 100 ppm [14] and this general toxicity analysis was carried out according to Nyamoita et al. method [17].

### 2.4. Data Analysis

The differences of seawater variables and bioactivity level at each site were analyzed by Kruskal-Wallis statistical analysis. Quantification of the relationship between seawater variables, local coral cover, and bioactivity level was analyzed by bivariate nonparametric Spearman analysis. A group of bioactivity levels had been created for the multivariate analysis, with activity higher than 50% categorized as “high,” activity between 30% and 50% categorized as “moderate,” and activity below 30% categorized as “low.” Multivariate Canonical Discriminant Analysis (CDA) was used to define the pattern of bioactivity level at the spatial competition variation of each sponge. One-Way Analysis of Similarities (ANOSIM) and Similarity Percentage (SIMPER) analysis had been applied to define the differences between the sponge spatial competition pattern at each bioactivity group (high, moderate, and low) and the factors contributed to the differences. Prior to multivariate analysis, the data of coral cover were log transformed and normalized. All of the statistical analyses had been done with Past Statistical Software v3.08 [18].

## 3. Results and Discussion

**Figure 2:**
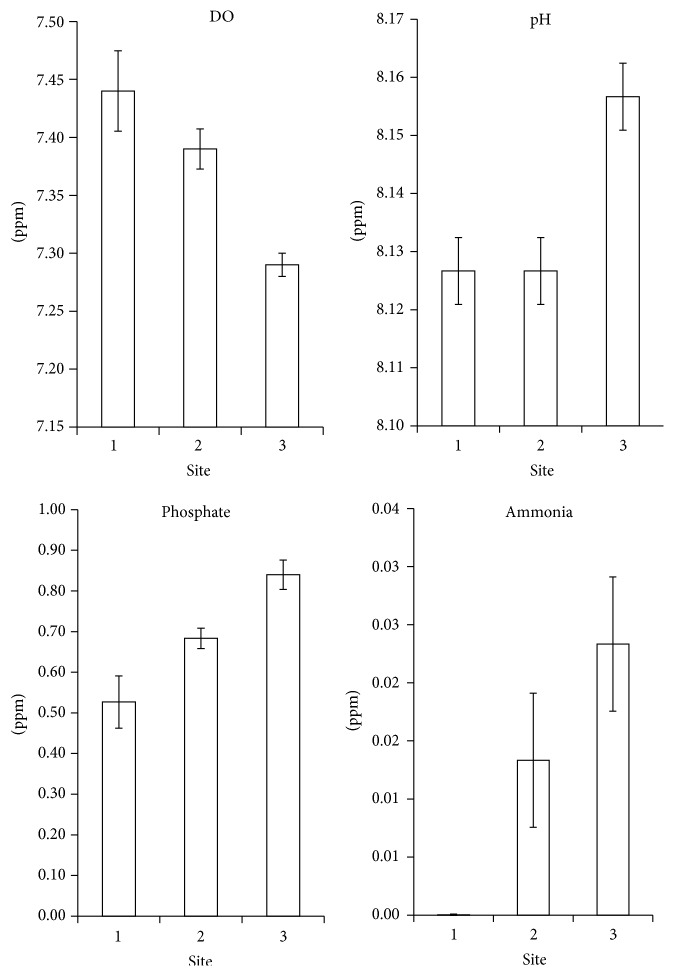
Average values of DO, pH, phosphate, and ammonia concentration in sampling sites (mean ± SD, with *n* = 6).

**Figure 3:**
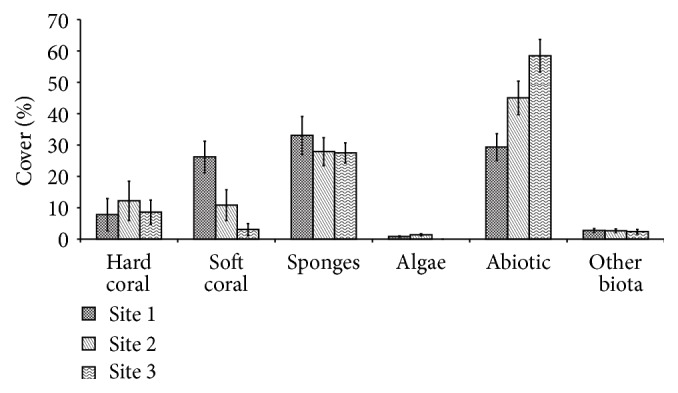
Average of coral cover in sampling sites at Hoga Island (mean ± SD, *n* = 3).

The water analysis results reflected the environmental condition at each site (Figure 2). A significant difference of DO, phosphate, and ammonia between sites was detected by Kruskal-Wallis nonparametric test (*P* < 0.05). Higher levels of phosphate and ammonia were detected in Site 3 compared to Sites 1 and 2. Nutrient contamination likely comes from domestic run-off and chemical fertilizers and therefore may serve as an indication of human activities pressures [19]. Meanwhile, DO in Site 1 is shown to be higher, as it is close to the sea wave reach and the reef breaks compared to Sites 2 and 3. The gradient impact of anthropogenic pressures from Site 3 to Site 1 was detected by an increment in abiotic cover in Site 3 compared to Sites 1 and 2 (Figure 3).

Bivariate Spearman correlation showed a strong and significant relation between abiotic cover and the level of phosphate (*R* = 0.767, *P* < 0.05) and ammonia (*R* = 0.833, *P* < 0.05). Development to support recreational facilities in the southern side, such as seaport near Site 3, and other various anthropogenic may contribute to the degradation of coral cover in this area. Moreover, near to the south is the Kaledupa Island, having one of the densest populations in this area. Visual observation noted enormous plastic wastes along the southern beach, which indicates anthropogenic run-off, and stresses the coral reef of Hoga Island. The detection of biological community shift at the pressures of natural or anthropogenic stressors can be analyzed by spatial or temporal design. Even though temporal analysis is more comprehensive and may be applied to long term changes to detect a shift within gradient environmental changes, spatial analysis can also be used to detect a biological community shift in abrupt changes in environmental quality. The design of spatial analysis to find variability in coral reef community had been done in many researches, such as in Florida Reefs Tract, USA, and Jakarta Bay, Indonesia [20, 21]. With spatial analysis by gradient distance to the center of human activities, this research found anthropogenic are the major cause of coral community shift in Hoga Island reef.

Sponges were found to be distributed in all sites. However, the sponges cover tends to be decreased from Site 1 to Site 3 and had a moderate negative correlation to abiotic cover variable (*R* = 0.41, *P* < 0.05). Other experiments also found the same results; sponges were usually distributed at high coral cover site and negatively correlated at sand/sediment and low coral cover site [22]. This may means that even sponge has resistance toward wide range of environmental stresses [23, 24]; the cover of sponge might be expected to decrease as anthropogenic pressures increase.

**Figure 4:**
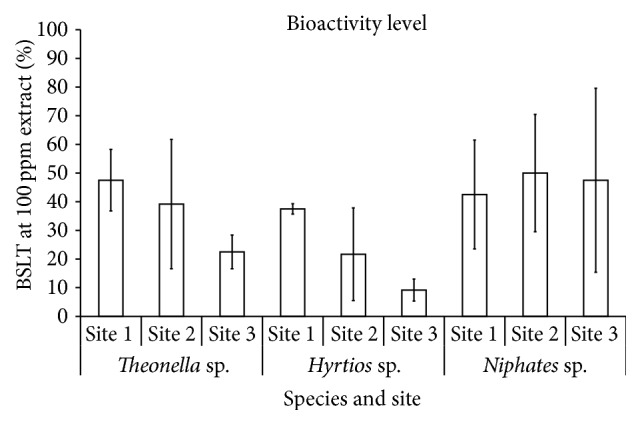
Bioactivity level by Brine Shrimp Lethality Test (BSLT) from each of the sponges at each sampling site in Hoga Island (mean ± SD, *n* = 4).

BSLT analysis found bioactivity level in all sponges to be higher in Site 1 and to be decreased as abiotic level increased (Figure 4). High deviation of bioactivity level from each sample was detected, even taken at the same sites. This may suggest that bioactivity of sponges relates to local spatial competition and depends on the needs to interact with local benthic environment. Statistical Spearman analysis showed that the level of bioactivity was negatively related to ammonia level (*R* = −0.683, *P* < 0.05) and abiotic cover (*R* = −0.900, *P* < 0.05). Both ammonia level and abiotic cover are key variables to detect increasing of anthropogenic pressures. Furthermore, this may support that interaction between biotic species is important as a trigger for sponges to produce their bioactive substances.

**Figure 5:**
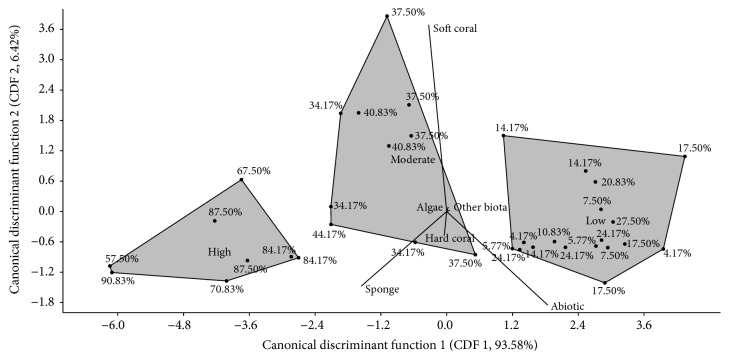
Score plot of CDA with sponges local coral cover as a variables to the sponges bioactivity, high (BSLT activity more than 50% at 100 ppm extract), moderate (BSLT activity between 30 and 50% at 100 ppm extract), and low (BSLT activity lower than 30% at 100 ppm extract).

The plot of Canonical Discriminant Analysis (CDA) strengthens the bivariate correlation analysis (Figure 5). CDA revealed the first function (93.91%) related to abiotic cover and the second function related to soft coral and hard coral cover (6.08%). One-way ANOSIM revealed the sponges' spatial competition from each bioactivity group (high, moderate, and low) was significantly different (*P* < 0.05) and SIMPER showed that abiotic cover (36.19%), sponges cover (28.34%), and soft coral cover (21.44%) were factors that contributed to the differences. CDA also showed the “high” group related to sponges cover, “moderate” group related to soft coral cover, and “low” group related to abiotic cover. This explanatory multivariate ordination analysis is explained in higher abiotic cover; sponges can be expected to produce lower amount of bioactive compounds. On the contrary, at the contact of benthic competition with other species such as with soft coral and other sponges, sponges will produce higher amount of bioactive compounds, to maintain or expand in benthic living space competition.

Based on the ecology of marine chemical defense, an invertebrate that is equipped with bioactive compounds, such as a sponge or a soft coral, may significantly dominate the spatial area compared with other organisms. The bioactive compounds are toxic chemicals which are used by these organisms to win spatial competition within benthic area of coral reefs [25–30]. From this analysis it can be concluded that the variance of live cover or abiotic cover in sponge environment may serve as an indication of bioactive level variability in sponges. These results supported that the spatial competition played important roles in the production of bioactive compounds from sponges.

## 4. Conclusion

Spatial competition played an important role in the production of bioactive compounds from sponges. The production of bioactive compound from sponges is highly dependent on the occurrence of other biotic species in the benthic community of coral reefs. Therefore, higher abiotic area, as an impact of the increasing anthropogenic pressures, will have a direct consequence to the lower production of bioactive compounds in sponges.
